# Insights into the Chemical Structure and Antioxidant Activity of Lignin Extracted from Bamboo by Acidic Deep Eutectic Solvents

**DOI:** 10.1021/acsomega.4c06259

**Published:** 2024-09-19

**Authors:** Kaiqin Song, Liping Yu, Shoulu Yang, Yan Cao, Lifen Li, Zhigang Wu, Hongtao Shi, Qiaorun Ma

**Affiliations:** † College of Forestry, Guizhou University, Guiyang 550025, China; ‡ Guizhou Academy of Forestry, Guiyang 550025, China; § School of Materials Science and Engineering, Guizhou Minzu University, Guiyang 550025, China; ∥ International Joint Research Center for Biomass Materials, Southwest Forestry University, Kunming 650224, China

## Abstract

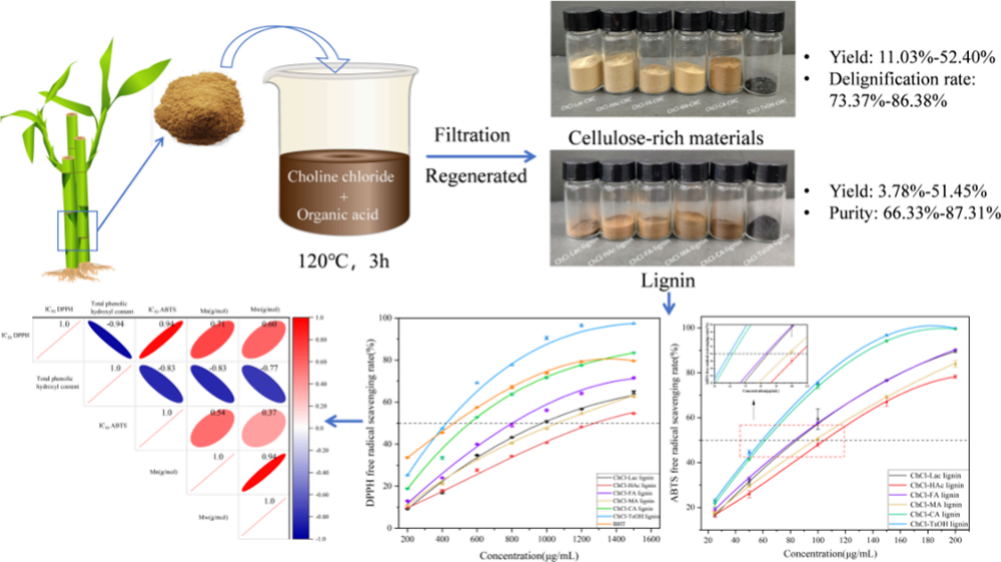

Deep eutectic solvents (DESs) composed of choline chloride as hydrogen bond acceptors (HBAs) and six organic acids as hydrogen bond donors (HBDs) were used to extract lignin from bamboo (*Phyllostachys edulis* (Carrière) J. Houz.). The structures of the DES-extracted lignin samples were analyzed by Fourier-transform infrared spectroscopy (FT-IR), UV–visible spectroscopy (UV–vis), thermogravimetric analysis (TG), and gel permeation chromatography (GPC) to investigate the relationship between the chemical structure of lignin and its antioxidant activity. The results showed that DES treatment removed a large portion of the lignin (73.37–86.38%) from bamboo, and the chemical structure of lignin was changed due to the use of different types of HBDs. The extracted lignin exhibited good UV–vis light shielding properties, thermal stability, and antioxidant activity. Moreover, the total phenolic hydroxyl content of lignins was positively correlated with their antioxidant activity, while the molecular weight of lignins was negatively correlated with their antioxidant activity. Notably, lignin extracted with choline chloride-*p*-toluenesulfonic acid had the highest phenolic hydroxyl content and lower molecular weight, showing the strongest antioxidant activity (IC_50 DPPH_ = 417.69 μg/mL, IC_50 ABTS_ = 58.62 μg/mL). This study confirms the high thermal stability, excellent antioxidant activity, and UV shielding properties of lignin extracted with choline chloride-organic acid DESs, suggesting its potential application in the fields of antioxidants and material modifiers.

## Introduction

Lignin is an amorphous natural polymer formed by oxidative coupling of phenylpropane structural units with many active functional groups, including methoxy, phenolic hydroxyl, alcohol hydroxyl, and carboxyl group. Numerous studies have demonstrated its antioxidative, antiultraviolet, and antibacterial properties, highlighting its potential applications in a variety of fields, such as food, cosmetic, and agriculture.^1−3^ However, the complexity of the molecular structure of lignin and the stability of its chemical properties hinder its effective separation and high-value utilization. Therefore, efficient separation and degradation of lignin are key factors in the development and utilization of lignocellulosic biomass resources. Currently, lignin extraction methods include a series of chemical, physical, and biological techniques, but these methods generally suffer from drawbacks, such as low extraction rates and harsh reaction conditions.

Deep eutectic solvents (DESs) are a eutectic mixture of hydrogen bond donor (HBD) and hydrogen bond acceptor (HBA) with a melting point lower than that of their individual components, which have the advantages of simple preparation, environmental friendliness, cost-effectiveness, and renewability. In recent years, DESs have received extensive research and attention in the field of wood fiber component separation and subsequent processing and utilization of the components.^4−6^ Some DESs have demonstrated excellent ability to solubilize or degrade lignin,^7^ while most DESs are unable to dissolve cellulose in lignocellulose. The extracted lignin retains most of the properties and activity of natural lignin.^8^ Based on the type of HBDs and HBAs, DESs can be classified as neutral, acidic, and alkaline. Tan et al.^9^ comparatively analyzed the extraction of lignin from oil palm hollow fruit shells by neutral, acidic, and alkaline DESs and found that acidic DESs performed better. Organic acid-based DESs demonstrate excellent lignin removal efficiency and have been applied to the efficient dissolution of lignin.^10^ The −OH and −COOH groups of the HBD in DESs provide active protons, which facilitate the cleavage of ether bonds and some C–C bonds during lignin extraction.^11^ Li et al.^12^ developed an acidic DES consisting of choline chloride and oxalic acid for bamboo delignification and found that acidic DESs enhanced the delignification process. Additionally, increasing the reaction temperature promoted the cleavage of the β-O-4 bonds, resulting in a higher content of phenolic–OH groups. Additionally, the interaction between HBA and HBD also facilitates the cleavage of unstable ether bonds between phenylpropanes units, resulting in the depolymerization and separation of lignin. The efficiency of lignin removal and the chemical structure of the obtained lignin are influenced by factors such as alkyl-chain length, hydroxyl group, carbonyl group, and carboxyl group of DESs.^13,14^ Currently, most DESs are prepared with choline chloride (ChCl) as the HBA, and the physical and chemical properties of ChCl-based DESs, including intermolecular hydrogen bonding, affect the extent of lignin removal. In addition, parameters such as the type of functional group or the carbon chain length in the HBD can negatively affect lignin removal. For instance, HBDs containing a carboxyl group exhibit greater lignin solubility compared to those containing hydroxyl and amino/amide groups.^13,15^

There are many functional groups in the lignin, including phenolic hydroxyl groups that confer antioxidant properties to scavenge oxygen free radicals and benzene ring structures with ultraviolet absorption capabilities. In recent years, lignin-based products have attracted much attention for their excellent antioxidant and UV-protective properties, which have attracted the interest in food, cosmetics, and polymeric materials industries.^16^ For instance, studies have shown that the addition of lignin to cosmetic formulations enhances the UV-blocking efficacy of sunscreens, with the sun protection factor improving with increasing lignin concentration.^17^ Moreover, the addition of lignin to a polymer can effectively retard photo- and thermal oxidation, prolonging the service life of the material and stabilizing its properties.^18^ However, the differences in chemical structures resulting from different treatments of DESs used to extract and modify lignin greatly affect the development and application of lignin-based products. Therefore, it is crucial to explore the relationship between the chemical structure of lignin and its antioxidant activity. Exploring new ways to utilize lignin in a high-value and functional way is of great theoretical significance and practical value to promote the development of the biomass industry and to achieve the goals of “carbon peak” and “carbon neutrality”.^19,20^

In recent years, the combination of choline chloride and organic acids is the most favorable DESs for the separation and extraction of lignin, which can be efficiently extracted lignin from plant fibers with high purity.^21^ In the present study, six kinds of acidic DESs composed of choline chloride with monocarboxylic acids (lactic acid, acetic acid, and formic acid), dicarboxylic acid (malic acid), tricarboxylic acid (citric acid), and Bro̷nsted acid (*p*-toluenesulfonic acid) were used to extract lignin from bamboo (*Phyllostachys pubescens*). Subsequently, the chemical structure, thermal stability, and relative molecular weight of the DES-extracted lignin were analyzed by FTIR, UV–vis, TG, and GPC, and the relationship between the chemical structure of the lignin and its antioxidant activity was explored to lay the foundation for the development of high-value lignin-based materials with antioxidant functionalities.

## Materials and Methods

### Materials

Lactic acid (analytical grade, 85.0% to 90.0%) and potassium persulfate (analytical grade, 99.5%) were purchased from Saranbo Technology Co., Ltd. Choline chloride (analytical grade, 98%), acetic acid (analytical grade, ACS, ≥99.7%), formic acid (ACS, ≥96%), malic acid (analytical grade, >99.0%), citric acid (analytical grade, 99.5%), *p*-toluenesulfonic acid monohydrate (analytical grade, ≥98.5%), methanol (chromatographic grade, ≥99.9%), 1,4-dioxane (ACS, ≥99.0%), 1,1-diphenyl-2-picrylhydrazine radical (DPPH) (97%), 2,2′-azinobis- (3-ethylbenzthiazoline-6-sulfonate) (ABTS) (98%), acetyl chloride (AR, 98%), glacial acetic acid (AR, 99.5%), gallic acid (99%), anhydrous sodium carbonate (≥99.8%), and other reagents were purchased from Shanghai Aladdin Biochemical Technology Co., Ltd. All materials were used directly without any treatment.

### Preparation of DESs

HBA and HBDs were mixed in a wide-mouthed bottle at a certain molar ratio (see Table 1 for details). The mixtures of ChCl-Lac, ChCl-HAc, and ChCl-FA were prepared at 80 °C for 30 min at 400 rpm to obtain a transparent solvent. ChCl-MA, ChCl-CA, and ChCl-TsOH were prepared at 100 °C, also at 400 rpm, for a duration of 1 to 2 h to form a transparent liquid. These synthesized DESs were subsequently cooled to room temperature and stored in a desiccator. It was observed that ChCl-MA transitioned into a solid phase after 2 to 3 days at room temperature, possibly due to the diminishing strength of the hydrogen bond formed between choline chloride and malic acid as the temperature dropped.^22^ Therefore, the DESs used in this experiment were prepared at the time of use. The pH values of the various DESs were measured at 20 °C using an acidimeter (PHS-3C). Each sample was tested three times, and the average value was recorded.

**Table 1:** Composition of the DESs

HBA	HBD	mole ratio	pH	label
choline chloride (ChCl)	lactic acid (Lac)	1:2	3.37 (0.06)	ChCl-Lac
	acetic acid (HAc)	1:2	1.10 (0.02)	ChCl-HAc
	formic acid (FA)	1:2	–0.04 (0.04)	ChCl-FA
	malic acid (MA)	1:2	–0.62 (0.05)	ChCl-MA
	citric acid (CA)	1:2	–1.43 (0.11)	ChCl-CA
	*p*-toluenesulfonic acid (TsOH)	1:1	–1.92 (0.08)	ChCl-TsOH

### Acidic DES Extraction of Bamboo Lignin

**Scheme 1:**
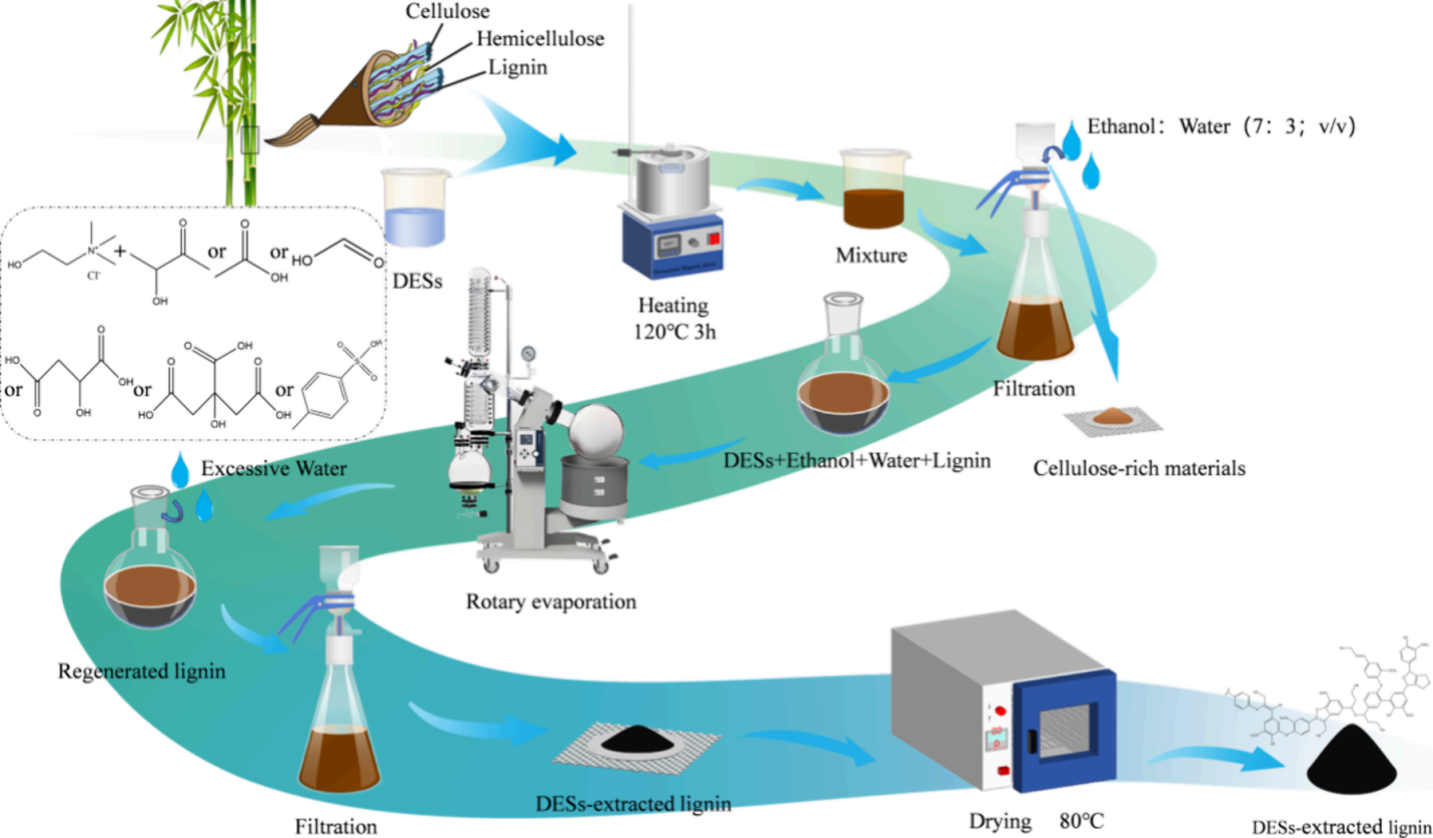
Extraction Route of Bamboo Lignin with DESs

Based on previous research with slight modifications,^23,24^ lignin was extracted from bamboo using acidic DESs, as presented in Scheme 1. We weighed approximately 1 g (with an accuracy of 0.0001 g) of bamboo powder and mixed it with the above-mentioned DESs. The reaction was carried out on a magnetic stirrer with a solid/liquid ratio of 1:20 g/mL, at an extraction temperature of 120 °C, and an extraction time of 3 h at 400 rpm. After the reaction period was over, the reaction mixture was removed and 20 mL of 70% ethanol (v/v). After the mixture was cooled to room temperature, the soluble and insoluble components of the DESs were separated by vacuum filtration. Then, 130 mL of 70% ethanol was added slowly in batches. Subsequently, the solid insoluble material was washed multiple times with distilled water to remove the residual DESs to obtain a cellulose-rich solid and lignin solution. Next, the residual ethanol in the lignin solution was removed by a rotary evaporator, and excess water was then added to precipitate the lignin. Finally, the precipitated lignin was dried in an electric, thermostatic drying oven for storage and further use.

### Determination the Chemical Composition of Bamboo and Cellulose-Rich Material

About 1 g of absolutely dry bamboo powder was weighed, and the yields of cellulose-rich materials and lignin after different DES treatments were calculated by a gravimetric method. The contents of cellulose, hemicellulose, and lignin in the raw bamboo materials as well as the lignin content in the cellulose-rich materials were quantitatively analyzed using reagent kits (Beijing Solebao Technology Co., Ltd.). The chemical composition of the DES-extracted lignin samples was analyzed according to the national standard GB/T 35818–2018^25^ and GB/T 10337–2008.^26^ The purity of the DES-extracted lignin samples was calculated from the contents of acid-insoluble lignin (AIL), acid-soluble lignin (ASL), and ash (ASH).^27^ The cellulose-rich material (CRM) yield, lignin yield, delignification rate, and purity of the lignins were calculated according to the following formulas:
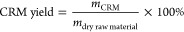
1where *m*_CRM_ is the mass of recovered CRM and *m*_dry raw material_ is the mass of dry raw material;
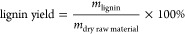
2where *m*_lignin_ is the mass of extracted lignin;
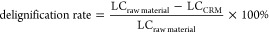
3where LC_raw material_ and LC_CRM_ are the lignin contents of raw material and CRM, respectively; and
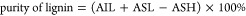
4

### Characterization of the DES-Extracted Lignin Samples

#### Infrared Spectral Analysis

FT-IR spectrometer (Nicolet iS20, Thermo Scientific, USA) was used to record the FTIR spectra of the DES-extracted lignin samples in the wavelength range of 4000 to 400 cm^–1^ with a resolution of 2 cm^–1^.

#### Ultraviolet–Visible Spectral Analysis

UV spectra of the DES-extracted lignin samples were analyzed by using a UV–vis spectrophotometer (UV-9000, Shanghai Yuan Analysis instrument). The lignin was dissolved in dioxane/water solution (9:1, v/v), and the spectra were scanned in the wavelength range of 230 to 600 nm. Before measurement, 90% of the dioxane/water solution was scanned at the same wavelength to establish a baseline.

#### Thermal Stability Analysis

The thermal stability of the DES-extracted lignin samples was tested by using a thermogravimetric analyzer (Netzsch, STA 2500). The samples were heated at a heating rate of 10 °C/min within a temperature range of 30 to 800 °C under the protection of high-purity nitrogen. Thermogravimetric curves and derivative thermogravimetric curves were recorded.

#### Molecular Weight Analysis

Changes in the molecular weight of lignin reflect the extent of its hydrolysis, polymerization, and condensation reactions. Prior to the determination of the molecular weight of lignin, the lignin samples were modified through acetylation to increase its solubility in tetrahydrofuran. Referring to the method in the reference,^28^ 0.5 g of lignin was mixed with 10 mL of acetylation reagent (acetyl chloride/glacial acetic acid, 1:4, v/v) and heated in water bath at 40 °C for 2 h. Afterward, the reaction solvent was removed by rotary evaporation, and the filter residue (acetylated lignin) was washed with distilled water until odorless of acidity. The recovered acetylated lignin samples were dried in a vacuum desiccator at 40 °C and then stored in a desiccator. The molecular weight of the acetylated lignin was analyzed using gel permeation chromatography (Waters 1525/Waters 2414) with tetrahydrofuran as the solvent.

#### Total Phenolic Hydroxyl Content of the DES-Extracted Lignin Samples

The total phenolic hydroxyl groups content in lignin was determined by the Folin–Ciocalteu method using gallic acid as a standard substance.^29^ For the test, 0.2 mL of gallic acid solutions of different concentrations (40, 80, 120, 160, 200, and 240 μg/mL) was mixed with 0.5 mL of Folin reagent, shaken, and reacted for 3 min. Afterward, 1 mL of 15% Na_2_CO_3_ solution was added to the mixed solution, diluted to 10 mL with water, and shaken in the dark for 2 h at room temperature. Finally, the absorbance of gallic acid solution was determined by UV–vis spectrophotometer within the concentration range of 40–240 μg/mL. A standard curve of gallic acid was plotted with the concentration as the horizontal axis and absorbance as the vertical axis.

Lignin solution with a concentration of 1 mg/mL in dioxane/water (90:10, v/v) was prepared, and 0.2 mL of this solution was taken and processed as described above to measure its absorbance at 760 nm. The phenolic hydroxyl content of lignin was calculated according to the gallic acid standard curve. The total phenolic hydroxyl content of the sample was expressed as gallic acid equivalent (GAE), i.e., mg GAE/100 mg lignin.

### Antioxidant Activity Analysis of DES-Extracted Lignin Samples

#### Analysis of Scavenging Ability of Lignin on DPPH Free Radicals

DPPH^·^ is a stable free radical capable of capturing or scavenging other free radicals. This stability is attributed to its electron-absorbing NO_2_ groups and the extensive π bond in its benzene ring. The scavenging reaction of DPPH^·^ can be detected due to its strong absorption at 517 nm, which gives the alcohol solution a characteristic purple color. When other free radical scavengers are present, they can pair with the unpaired electrons of DPPH^·^, causing gradual discoloration of the solution. The extent of this discoloration is quantitatively related to the number of electrons received by DPPH^·^.^30^ In this experiment, lignin solutions of different concentrations (0.2–1.5 mg/mL) were prepared using dioxane/water solution (9:1, v/v). 0.1 mL portion of the above-prepared lignin solution was mixed with 3.9 mL of DPPH/methanol solution at a concentration of 0.0024 mg/100 mL. The mixture was kept in the dark for 30 min, after which the absorbance (A) was measured at 517 nm using a UV spectrophotometer (UV-9000, Shanghai Yuanxi Instrument). Additionally, the absorbance (*B*_1_) of the lignin methanol solution without DPPH, also kept in the dark for 30 min, was measured. The scavenging rate of DPPH^·^ was calculated according to formula 5. A higher scavenging rate indicates a stronger antioxidant capacity of lignin. Each experiment group was repeated three times, and the average value was taken.
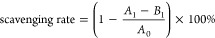
5

In the formula, *A*_0_ is the absorbance of the control group, *A*_1_ is the absorbance of the solution after the addition of lignin samples, and *B*_1_ is the absorbance of the lignin methanol solution.

#### Analysis of the Scavenging Ability of Lignin to ABTS Free Radical

The scavenging of ABTS cationic free radicals (ABTS^+·^) is mainly due to the transfer of electrons or protons. It is considered that the ability of lignin to scavenge ABTS^+·^ may be related to the content of phenolic hydroxyl and carboxyl groups.^31,32^ In this experiment, 7.4 mM ABTS solution was reacted with 2.6 mM potassium persulfate, and the mixture was placed in the dark at room temperature for 12–16 h to generate ABTS^+·^. A mixture of 50 mL of methanol and 1 mL of the ABTS^+·^ solution was prepared to obtain an absorbance of 0.70 ± 0.02 at 734 nm. Different concentrations of lignin sample solution (150 μL) were mixed with either the ABTS/methanol solution or a methanol solution (1950 μL). The absorbance at 734 nm was recorded as *A*_1_ and *B*_1_ after reaction for 2 h at room temperature in a dark environment. The scavenging rate of ABTS^+·^ was calculated by using formula 6. Each experiment was repeated three times, and the average value was taken.
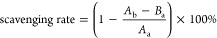
6

In the formula, *A*_a_ is the absorbance of the control group, *A*_b_ is the absorbance of the solution after the addition of lignin samples, and *B*_a_ is the absorbance of the lignin methanol solution.

### Statistical Analysis

Statistical analyses were conducted with SPSS version 25.0, significance was set at *p* < 0.5 for all analyses.

## Results and Discussion

### Effects of DES Type on Bamboo Lignin Extraction

Recent studies on the pretreatment of lignocellulosic biomass with DESs have attracted widespread attention, especially those DESs based on organic acid as HBDs, which have demonstrated excellent lignin removal ability.^33^ Untreated bamboo feedstock contains 45.44% cellulose, 26.17% hemicellulose, 26.10% lignin, and a small amount (2.30%) of ash and other substances.

**Figure 1:**
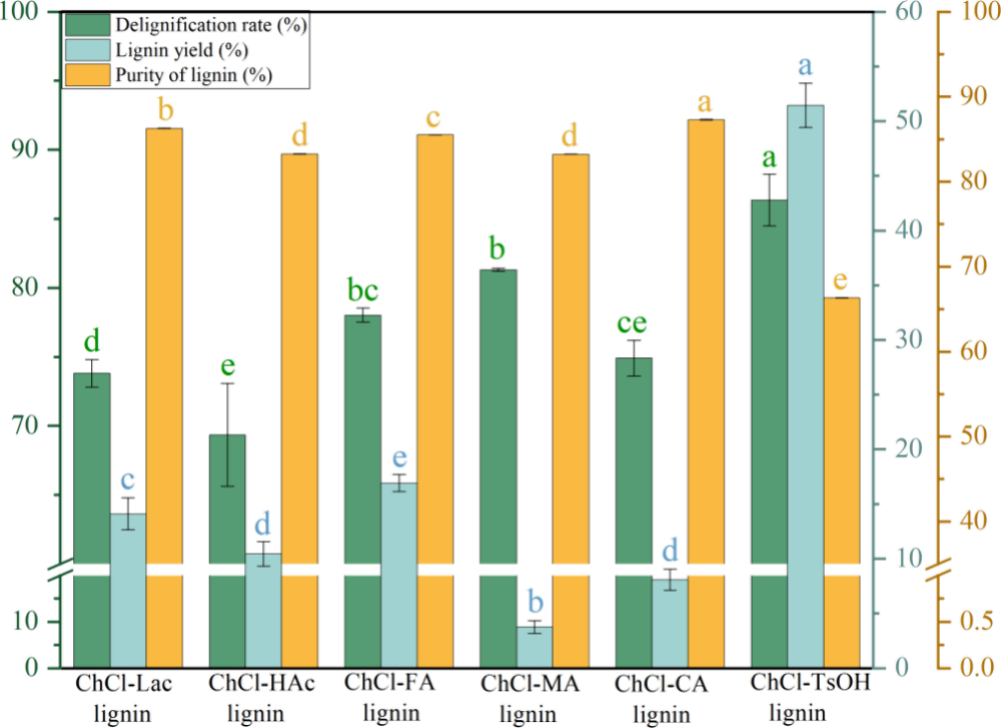
Delignification rate, lignin yield, and purity in different DES treatments. Different lowercase letters indicated significant differences (*n* = 3, *p* < 0.05).

Six kinds of DESs were synthesized with choline chloride as the HBA and various organic acids (lactic acid, acetic acid, formic acid, malic acid, citric acid, and *p*-toluenesulfonic acid) as the HBDs. The effects of these DESs on the cellulose-rich material yield, lignin yield, delignification rate, and purity of the DES-extracted lignin samples were analyzed, and the results are shown in Table 2 and Figure 1. It was observed that DES treatment resulted in considerable lignin removal (ranging from 73.37 to 86.38%), with efficiency greatly influenced by the type of HBDs in the DESs. The highest delignification rate was obtained with ChCl-TsOH (86.38%) followed by ChCl-Lac, ChCl-FA, ChCl-HAc, ChCl-CA, and ChCl-MA, and this finding agrees well with Chen et al., who showed that the monocarboxylic acid HBD possessed the optimal extraction capacity compared with the di- and tricarboxylic acids.^34^ Lignin removal primarily occurred through the cleavage of ether or ester bonds between lignin and polysaccharides (cellulose and hemicellulose), catalyzed by protons (H^+^) from the dissociation of HBDs in the DESs.^8^ However, consistent with the observations by Hou et al.,^35^ no clear correlation was observed between the pH values of the DESs and their lignin extraction capacities. Additionally, DESs degraded lignin by breaking C–C bonds (to a lesser extent) and extensively breaking C–O–C bonds in the side chains of lignin’s phenyl rings, thereby achieving the purpose of effectively separating lignin from carbohydrate components.^35−38^

**Table 2:** Effect of DES Treatment on Delignification of Bamboo

DESs		ChCl-Lac	ChCl-HAc	ChCl-FA	ChCl-MA	ChCl-CA	ChCl-TsOH
yield of CRM (%)		51.39	52.10	50.19	52.40	50.98	11.03
residual lignin in cellulose-rich material (%)		5.75	6.84	6.45	6.95	6.54	3.55
lignin yield (%)		14.12	10.47	16.94	3.78	8.10	51.45
composition of the DES-extracted lignin samples	AIL (%)	83.55	80.79	82.14	81.55	84.68	64.94
	ASL (%)	3.12	2.96	3.6	2.77	3.05	3.84
	purity of lignin (%)	86.28	83.26	85.53	83.24	87.31	66.33
	ash (%)	0.39	0.49	0.21	1.03	0.41	2.45
delignification rate (%)		77.95	73.78	75.28	73.37	74.92	86.38

*p*-Toluenesulfonic acid, acting as a Lewis acid, efficiently breaks the C–O–C bonds between lignin phenylpropane structural units, forming hydroxyl groups.^39,40^ However, precise control of processing conditions is necessary during Lewis acid-catalyzed lignin dissociation to prevent damage and degradation of the carbohydrate components.^40^ Several studies have highlighted the efficacy of choline chloride-lactic acid in lignin removal, attributed to its structure and composition, including functional groups.^35,36^ This DES disrupts interactions between lignin and carbohydrates and promotes the cleavage of the β-O-4 bond in lignin to some extent, thereby decreasing the molecular weight of lignin and increasing the content of functional groups (e.g., phenolic and alcoholic hydroxyl groups).^37,38^ The DESs formed by formic acid and choline chloride exhibited strong hydrogen bonding effects, and the acidic properties of formic acid facilitate the hydrolysis of cell wall components, resulting in a higher lignin extraction rate. However, the toxicity and corrosiveness of formic acid limit its applicability.^41^

It was found that the purity of lignins extracted with the other five DESs was relatively high (83.24%∼87.31%) except for ChCl-TsOH lignin, which had a lower purity of 66.33%. In the experiment, the cellulose-rich material separated by ChCl-TsOH appeared as carbon granular with a reduced yield, possibly due to the strong acidity of *p*-toluenesulfonic acid causing some degree of carbonization of the carbohydrate components in the bamboo material and the formation of small molecular substances after treatment at 120 °C for 3 h. Studies have indicated that during DES processing, pseudolignin with structural characteristics similar to natural lignin may form, containing phenolic and furan oxygen-containing groups, exhibiting characteristics of lignin.^42^ Therefore, ChCl-TsOH lignin may contain a certain amount of carbonized cellulose, hemicellulose, and other impurities, resulting in lower purity but higher yield. This was influenced by various factors during the extraction process, including solvent composition, ratios, reaction conditions, etc.^9,43^

### Infrared Spectra of the DES-Extracted Lignin Samples

**Figure 2:**
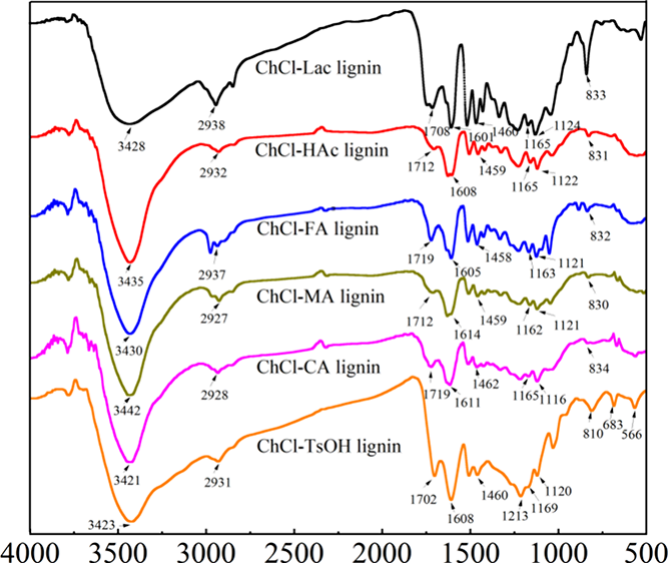
FTIR spectrum of DES-extracted lignins.

The chemical structure of the lignins was analyzed by infrared spectroscopy, as shown in Figure 2. It can be seen that all lignins exhibited similar spectral features, indicating that the extracted lignins had similar chemical structures, and retained the original aromatic compound structure. However, it can be seen that there were some differences in the intensity of the characteristic peaks of the different types of lignin, indicating that the content of certain functional groups in lignins was influenced by the extraction solvent.

It can be found from Figure 2 that all DES-extracted lignins appeared with a broad hydroxyl group band near 3425 cm^–1^, indicating the presence of abundant phenolic and aliphatic hydroxyl groups. Nevertheless, there were differences in the intensity of these absorption peaks across the six DES lignins. Specifically, ChCl-Lac lignin had the lowest peak intensity, possibly due to the different composition of DESs resulting in different degrees of ether bond cleavage. The strong peaks observed between 2931 and 2833 cm^–1^ were attributed to the C–H stretching vibrations of methyl or methylene groups.^44^ The absorption peak near 1700–1719 cm^–1^ belonged to the double-bond stretching vibration of nonconjugated ketones, carbonyls, and esters. ChCl-Lac lignin and ChCl-FA lignin showed higher peak intensities in this region, potentially indicating the occurrence of esterification, while the higher peak intensity at 1702 cm^–1^ in ChCl-TsOH lignin may be due to the formation of Hibbert’s ketones during lignin processing.^45^

The aromatic skeleton vibration of lignin, typically observed around 1510 cm^–1^, was clearly shown in all six DES-extracted lignins, indicating that lignins retained the aromatic skeleton structure of the original lignin. The absorption peaks appearing at 1270 and 1215 cm^–1^ corresponded to the C–O and C=O stretching vibrations in the guaiacyl (G) unit, whereas the absorption peaks near 1120 and 830 cm^–1^ were attributed to the C–H stretching absorption in the syringyl (S) unit. The absorption peak near 1160 cm^–1^ was assigned to the stretching vibration of the lignin ether bond C–O–C. It can be found that all DES-extracted lignins exhibited absorption peaks at this point, with ChCl-TsOH lignin showing a certain degree of red-shift to 1169 cm^–1^ compared to the other DES-extracted lignins. This shift might be due to the more severe disruption of the aromatic ether bonds (especially the β-O-4 ether bonds) in the lignin structure. In addition, the obvious absorption peaks at 633 and 566 cm^–1^ in ChCl-TsOH lignin, which may be due to the stretching vibration of the sulfate group (SO_3_), suggest that sulfonation of lignin might have occurred during the treatment of bamboo with ChCl-TsOH.^46,47^

### Ultraviolet–Visible Spectra of the DES-Extracted Lignin Samples

**Figure 3:**
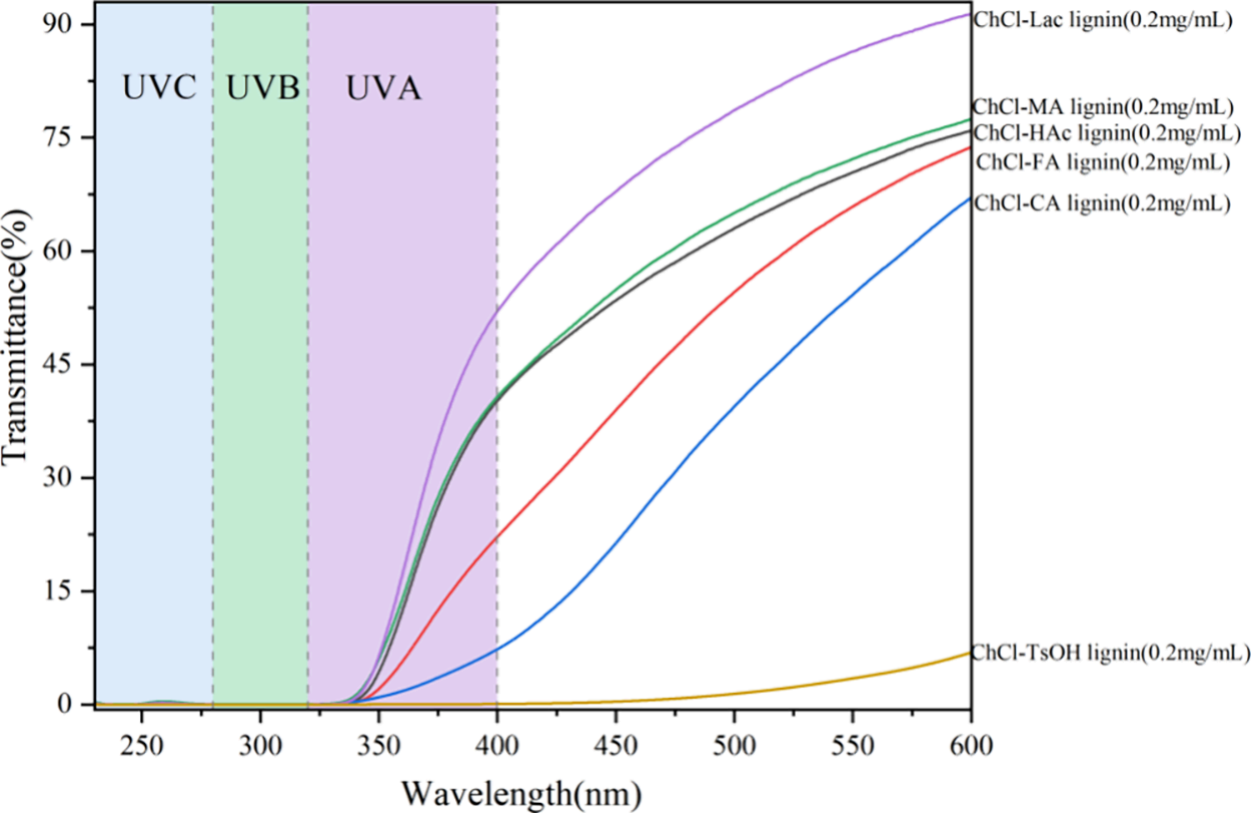
Ultraviolet–visible light transmission spectrum of the DES-extracted lignins.

**Figure 4:**
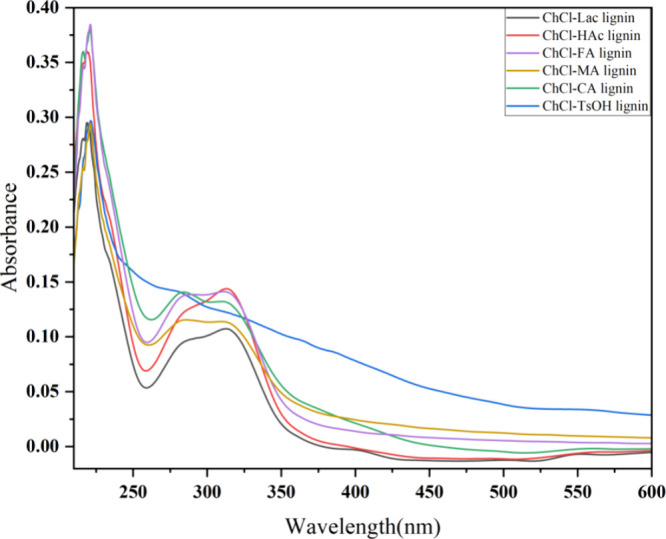
Ultraviolet–visible absorption spectrum of DES-extracted lignins.

Ultraviolet (UV) radiation is part of sunlight and can be categorized by wavelength into UVA (315–400 nm), UVB (280–315 nm), and UVC (100–280 nm). Studies have found that excessive exposure to UVA and UVB radiation is associated with DNA damage, premature skin aging, inflammation, and skin cancer.^48^ Additionally, UVA and UVB radiation can cause aging and discoloration of polymer materials.^49^ Lignin is a natural polymer rich in aromatic structures containing various UV-absorption functional groups such as ketones, phenolics, and chromophoric groups, which endow it with broad-spectrum and highly efficient UV absorption ability. The UV–visible transmission and absorption spectra of six DES-extracted lignins were analyzed, and the results are shown in Figures 3 and 4.

Figure 3 shows the UV–visible light transmission spectra of the DES-extracted lignins in the range of 230 to 600 nm. It can be seen that all lignins showed excellent UV shielding ability in both the UVC and UVB regions. In the UVA region, ChCl-TsOH lignin had the best UV shielding effect, with a transmittance of only 0.074% at 400 nm followed by lignin extracted using ChCl-CA (7.29% at 400 nm), ChCl-FA (22.12% at 400 nm), and ChCl-HAc and ChCl-MA (40.20% and 40.73%, respectively) while ChCl-Lac lignin showed the worst effective UV shielding, with a transmittance of 52.03% at 400 nm. Overall, the lignins extracted by using the six DESs demonstrated excellent UV shielding effects.

The shielding trend of the six DES-extracted lignins in the visible light range (400–600 nm) was similar to that in the UVA region. The high shielding effect of lignin in the visible region may be related to the presence of aryl, phenolic hydroxyl, ketone, carboxyl, and other chromophoric groups in lignin.^50^ Notably, ChCl-TsOH lignin also showed the lowest transmittance at 600 nm at only 6.88%. This may be due to the retention of pseudolignin formed by the degradation of the carbohydrate components after carbonization, in addition to the phenyl, hydroxyl, ketone, and other groups inherent to lignin itself. In 2022, Aarum et al.^51^ successfully separated pseudolignin from α-cellulose using DESs and found that pseudolignin is primarily produced from holocellulose, including glucose, xylose, and cellulose, and is enriched with carbonyl, carboxyl, aromatic, methoxy, and aliphatic in functional groups. The presence of pseudolignin interferes with the structural characterization of lignin.^52^ Overall, all six DES-extracted lignins exhibited notable UV–visible light shielding properties, with ChCl-TsOH lignin showing the strongest performance, making it a promising candidate for applications in UV shielding composites and sunscreens.

The UV absorption spectra of DES-extracted lignins in the range of 230 to 600 nm are shown in Figure 4. The characteristic benzene ring absorption band of lignin appeared at 280 nm, attributed to the π → π* electron transition of the aromatic ring. It can be observed that the absorption peak of lignin extracted using ChCl-Lac, ChCl-HAc, ChCl-FA, ChCl-MA, and ChCl-CA undergoes a red-shift to around 283 nm. This shift likely indicates a higher proportion of G-type molecular structure in the lignin.^53^ Additionally, ChCl-TsOH lignin showed a higher absorbance at 283 nm compared to other DES-extracted lignins, suggesting the presence of more guaiacyl compounds.^54^ The weak absorption peak near 318 nm corresponded to the n → π* electronic transition of the carbonyl (C_α_=O) bond conjugated to the aromatic ring, as well as the π → π* electronic transition of C_α_=C_β_ linkages, which may be related to the nonconjugated phenolic units in the lignin structure, such as ferulic acid, *p*-coumaric acid, or hydroxycinnamic acid.^55−57^

### Thermal Stability of the DES-Extracted Lignin Samples

**Figure 5:**
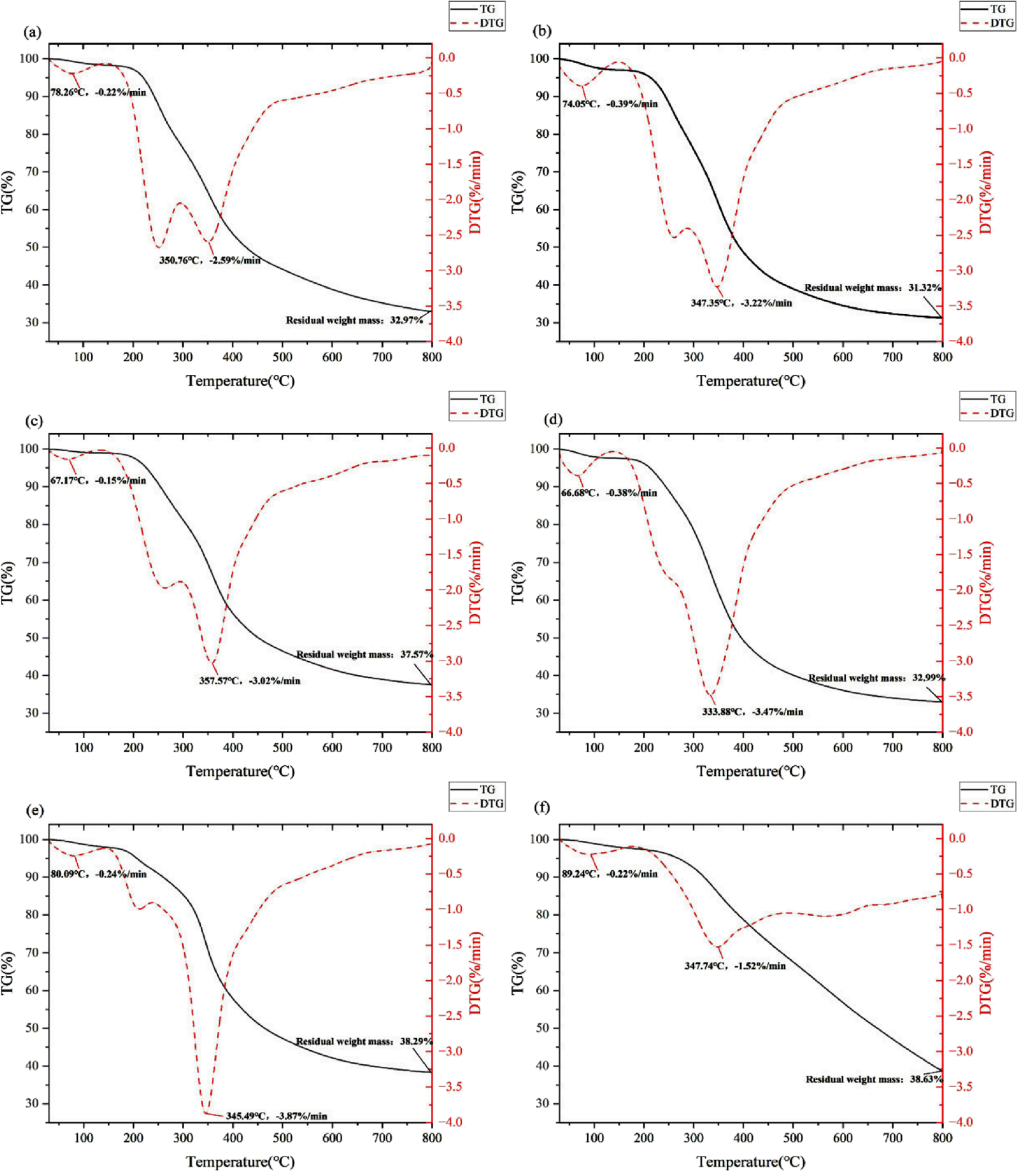
TG curve and DTG curve of the lignins extracted by DESs, where (a–f) are the TG curve and DTG curve of lignin extracted by ChCl-Lac, ChCl-HAc, ChCl-FA, ChCl-MA, ChCl-CA, and ChCl-TsOH, respectively.

Exploring the thermal properties of lignin helps us to understand the relationship between its structure and properties. In this study, the thermal stability of the six DES-extracted lignins was evaluated using a thermogravimetric (TG) analyzer in the temperature range of 30 to 800 °C. The TG curves and their first-order derivative (DTG) curves of the lignins are shown in Figure 5, and the corresponding thermal degradation characteristic data are presented in Table 3.

**Table 3:** Thermogravimetric Characteristic Data of the DES-Extracted Lignins^a^

	initial stage	first stage		second stage		
lignin type	*T*_0_/°C	*T*_p1_(°C)	degradation rate(%/min)	*T*_p2_(°C)	degradation rate(%/min)	carbon residue rate at 800 °C (%)
ChCl-Lac lignin	30.46	78.26	0.22	350.76	2.59	32.97
ChCl-HAc lignin	29.25	74.05	0.39	347.35	3.22	31.32
ChCl-FA lignin	29.37	67.17	0.15	357.57	3.02	37.57
ChCl-MA lignin	29.18	66.68	0.38	333.88	3.47	32.99
ChCl-CA lignin	29.09	80.09	0.24	345.49	3.87	38.29
ChCl-TsOH lignin	30.14	89.24	0.22	347.74	1.52	38.63

a *T*_0_/°C initial decomposition temperature; *T*_p1_/°C first stage decomposition fastest temperature; *T*_p2_/°C second stage decomposition fastest temperature.

It can be found that the thermal degradation of lignins can be divided into three stages. First, the initial decomposition stage spanned from 30 to 150 °C. The reduction of lignin mass during this stage is mainly caused by the evaporation of residual water in lignin and the volatilization of some small molecular weight substances. In this stage, the weight loss rate of the six DES-extracted lignins was less than 5%.

The second stage, which ranged from 150 to 500 °C, is characterized by a significant decrease in lignin mass. This mass loss is mainly due to the cleavage of chemical bonds (C–O and C–C in the aromatic ether structure) and the oxidation of side chains (carbonylation, carboxylation, or dehydrogenation).^58^ Among the six lignins, ChCl-FA lignin showed the highest temperature (*T*_p2_, 357.57 °C) corresponding to the highest rate of weight loss followed by ChCl-Lac lignin (350.76 °C), ChCl-TsOH lignin (347.74 °C), ChCl-HAc lignin (347.35 °C), ChCl-CA lignin (345.49 °C), and ChCl-MA lignin (333.68 °C). During this stage, all lignins except ChCl-TsOH lignin showed a shoulder peak. Especially, ChCl-Lac lignin showed a low-temperature shoulder peak at 252 °C with a weight loss rate of 2.67%/min, indicating that there were thermally instability regions in this lignin,^59^ similar to the thermal degradation behavior of lignins extracted using choline chloride-formic acid and choline chloride-lactic acid studied by Li et al.^60^ The *T*_p2_ of ChCl-TsOH lignin was 347.74 °C, similar to the other DES-extracted lignins, but its degradation rate (1.52%) was significantly lower, indicating that ChCl-TsOH lignin had the best thermal stability in this region.

In the temperature range from 500 to 800 °C, the weight loss rate of DES-extracted lignins slowed down, mainly involving the cleavage of the benzene ring C–C bond and dimethoxy group in the lignin skeleton structure^61^ as well as the degradation of branched and aromatic structure.^60,62^ As the temperature increased, part of lignin ultimately degraded into small molecules such as CO_2_, CO, H_2_O, etc.^63^

At 800 °C, the residual weight of lignin ranged from 31.32% to 38.63%. A higher residual weight indicates a greater presence of polycondensation structures in lignin, representing higher thermal stability.^64^ Among the six lignins studied, ChCl-TsOH lignin had the highest char yield (38.63%) followed by ChCl-CA lignin (38.29%), with ChCl-HAc lignin exhibiting the lowest char yield (31.32%).

### Molecular Weight of the DES-Extracted Lignin Samples

**Figure 6:**
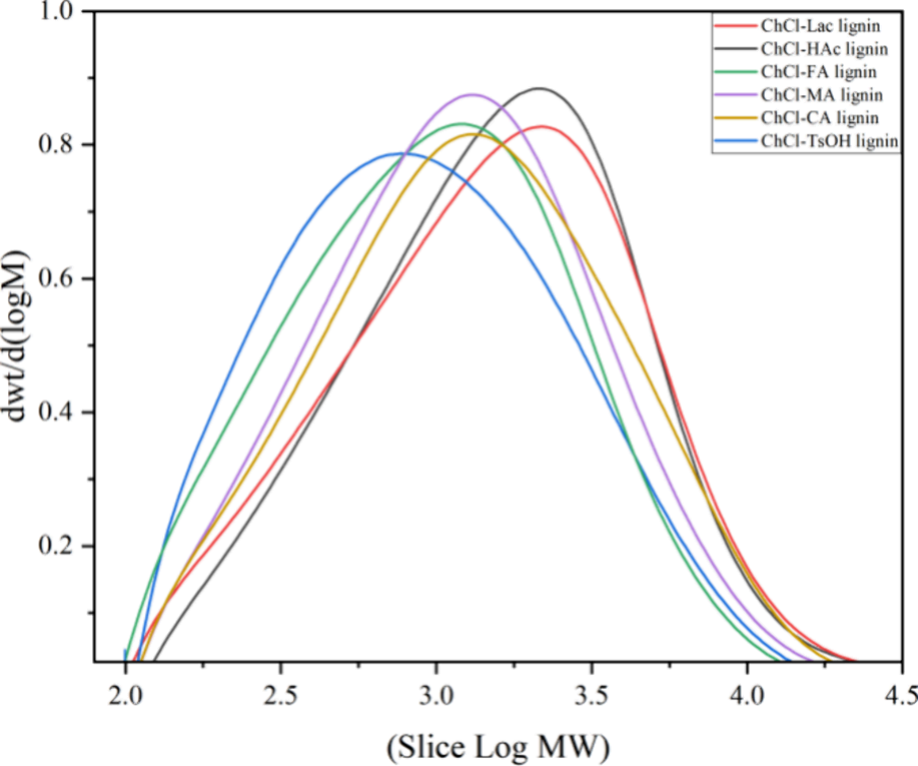
Molecular weight distribution of the DES-extracted lignins.

The relative molecular weights of the six acetylated DES-extracted lignins were determined by using gel permeation chromatography (GPC). The number-average molecular weight (*M*_n_), weight-average molecular weight (*M*_w_), and dispersion index (PDI, *M*_n_/*M*_w_) of the lignin samples are shown in Figure 6 and Table 4. It was observed that the *M*_n_ and *M*_w_ values of the lignins exhibited a relatively narrow range, ranging from 572 to 938 g/mol and from 1590 to 2643 g/mol, respectively. Additionally, the PDI values were low (2.66 to 3.12), indicating a narrow molecular weight distribution. Especially, ChCl-TsOH lignin had the lowest *M*_n_, *M*_w_, and PDI among the six DES-extracted lignins, suggesting that it had a smaller molecular weight and a more homogeneous molecular structure compared to the other samples.

**Table 4:** Molecular Weight of the DES-Extracted Lignins

lignin type	*M*_n_ (g/mol)	*M*_w_ (g/mol)	PDI
ChCl-Lac lignin	846	2643	3.12
ChCl-HAc lignin	938	2573	2.74
ChCl-FA lignin	601	1599	2.66
ChCl-MA lignin	736	1957	2.66
ChCl-CA lignin	774	2258	2.92
ChCl-TsOH lignin	572	1590	2.78

### Phenolic Hydroxyl Content of the DES-Extracted Lignin Samples

**Figure 7:**
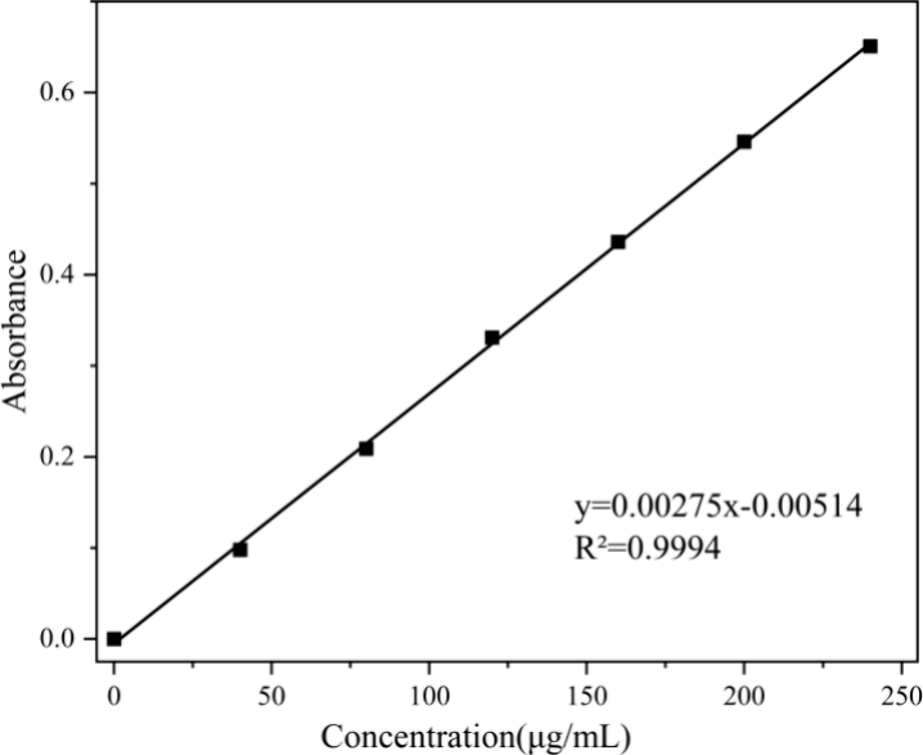
Relationship between gallic acid concentration and absorbance value

The phenolic hydroxyl content of the DES-extracted lignins was determined using the Folin–Ciocalteu method. A standard curve relating the gallic acid concentration to absorbance was constructed at 760 nm using a UV–vis spectrophotometer. The curve depicted a linear relationship between gallic acid concentration (abscissa) and absorbance (ordinate) as shown in Figure 7. The resulting regression equation was *y* = 0.00275*x* – 0.00514, with an *R*^2^ value of 0.9994, indicating a highly significant correlation. This suggests that there was a good linear relationship between gallic acid concentration and absorbance within the range of 40–240 μg/mL. Therefore, the gallic acid standard curve can be used to calculate the total phenolic hydroxyl content of lignin.

**Figure 8:**
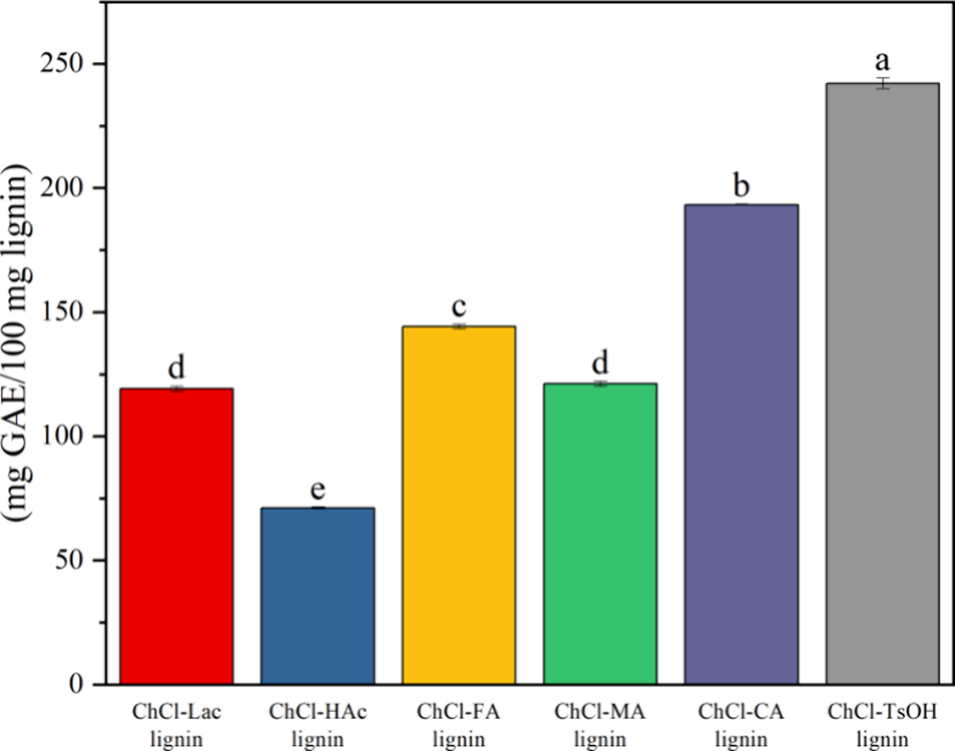
Total phenolic hydroxyl content of the DES-extracted lignins. Different letters (a–e) indicate values are significantly different (*n* = 3, *p* < 0.05).

The total phenolic hydroxyl content of DES-extracted lignins is shown in Figure 8. As can be seen from the figure, ChCl-TsOH lignin had the highest total phenolic hydroxyl content at 242.11 mg GEA/100 mg lignin followed by ChCl-CA lignin (193.26 mg GEA/100 mg lignin), ChCl- FA lignin (144.17 mg GEA/100 mg lignin), ChCl-MA lignin (121.14 mg GEA/100 mg lignin), and ChCl-Lac lignin (119.08 mg GEA/100 mg lignin), while ChCl-HAc lignin had the lowest total phenolic hydroxyl content, measuring only 71.08 mg GEA/100 mg lignin. By combining the results of previous infrared and thermogravimetric analyses along with the results of earlier studies,^39,60^ the high phenolic hydroxyl content of ChCl-TsOH lignin may be due to the destruction of more β-O-4 ether bonds between lignin structural units by choline chloride-*p*-toluenesulfonic acid during the extraction process. In contrast, the phenolic hydroxyl content of ChCl-Lac lignin and ChCl-HAc lignin was the lowest, possibly due to esterification reactions occurring during lignin extraction, which resulted in a lower residual phenolic hydroxyl content in the lignin.

### Antioxidant Activity of the DES-Extracted Lignin Samples

**Figure 9:**
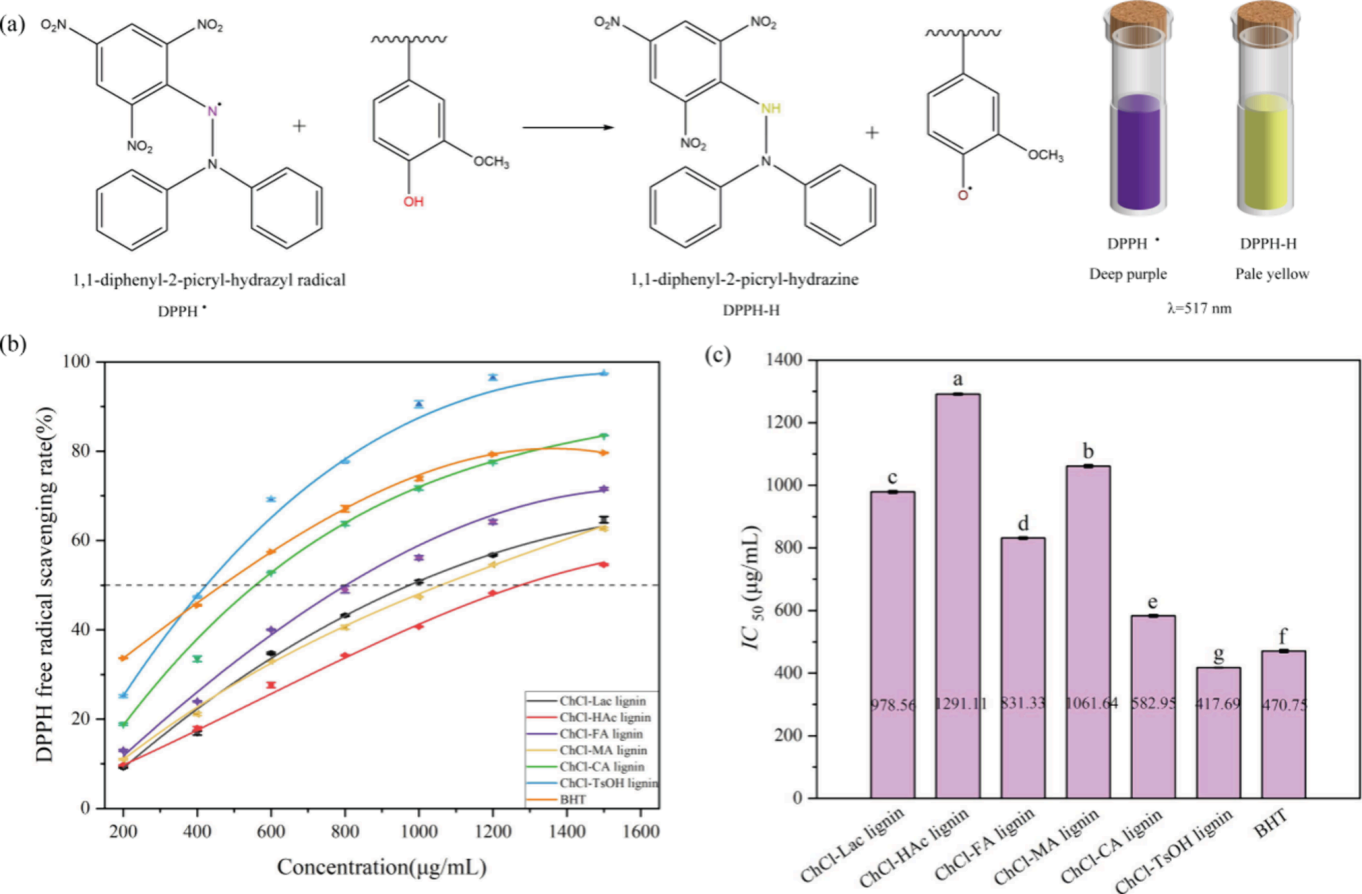
(a) Schematic of DPPH free radical scavenging by lignin, (b) scavenging effect of DES-extracted lignins and BHT on DPPH free radical, and (c) IC_50_ values of the DES-extracted lignins for DPPH radical scavenging activity. Data are expressed as means ± SD (*n* = 3). Different letters (a–g) indicate values are significantly different (*p* < 0.05).

**Figure 10:**
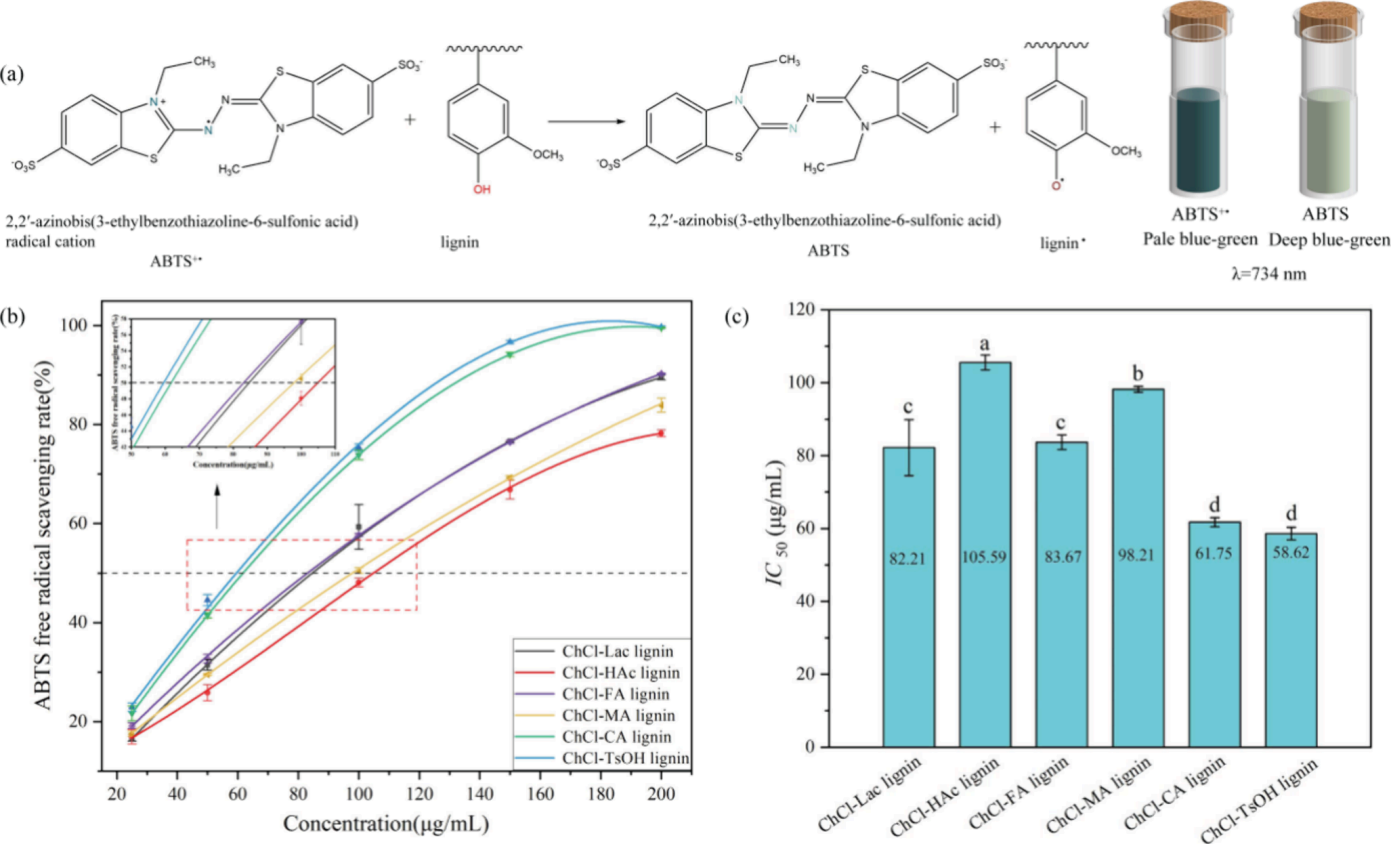
(a) Schematic of ABTS free radical scavenging by lignin, (b) scavenging effect of DES-extracted lignins on ABTS radical, and (c) IC_50_ values of the DES-extracted lignins for ABTS radical scavenging activity. Data are expressed as means ± SD (*n* = 3). Different letters (a-d) indicate values are significantly different (*p* < 0.05).

The antioxidant activity of the DES-extracted lignin samples was evaluated by using DPPH and ABTS radical scavenging assays, and the results are shown in Figures 9 and 10. It was found that all DES-extracted lignins were capable of scavenging DPPH^·^, with the scavenging rate increasing with an increase in lignin concentration. Differences were observed in the DPPH^·^ scavenging rates among different types of DES-extracted lignins. Among them, ChCl-TsOH lignin exhibited the strongest scavenging ability, surpassing the commercial antioxidant butylated hydroxytoluene (BHT) at a concentration of 400 μg/mL, with a scavenging rate of 47.41% compared to 45.48% for BHT. Especially encouraging was the excellent performance of ChCl-TsOH lignin at a concentration of 1200 μg/mL, achieving a high scavenging rate of 96.49%, compared to 79.29% for BHT.

The IC_50 DPPH_ value refers to the lignin concentration at which 50% of DPPH^·^ was scavenged. The smaller the value, the stronger the ability of lignin to scavenge DPPH^·^. Among the six DES-extracted lignins, ChCl-TsOH lignin showed the highest activity with an IC_50 DPPH_ of 417.69 μg/mL, followed by ChCl-CA lignin (IC_50 DPPH_ of 582.95 μg/mL), ChCl-FA lignin (IC_50 DPPH_ of 831.33 μg/mL), ChCl-Lac lignin (IC_50 DPPH_ of 978.56 μg/mL), ChCl-MA lignin (IC_50 DPPH_ of 1061.64 μg/mL), and ChCl-HAc lignin (IC_50 DPPH_ of 1291.11 μg/mL).

Similar trends were observed in the ABTS^+·^ scavenging assay; overall, the scavenging ability of lignin increased with concentration. At an optimal concentration of 200 μg/mL, the ABTS^+·^ scavenging rates ranged from 78.22% to 99.74%. However, the relationship between the lignin concentration and ABTS^+·^ scavenging ability was not entirely linear. At higher concentrations, the ABTS^+·^ scavenging rate of ChCl-TsOH and ChCl-CA lignin showed a plateau, which may be due to factors such as saturation of the lignin concentration, intermolecular interactions, solvent effects, and other undefined chemical reactions that may slow down the scavenging rate of ABTS^+·^.^65^ The IC_50 ABTS_ values of DES-extracted lignins ranged from 58.62 to 105.59 μg/mL, with ChCl-TsOH lignin exhibiting the lowest IC_50 ABTS_ value and ChCl-HAc lignin the highest. Therefore, these results suggested that DES-extracted bamboo lignin exhibited good antioxidant activity, which is expected to be applied in industries such as food, cosmetics, and polymer.^66,67^

**Figure 11:**
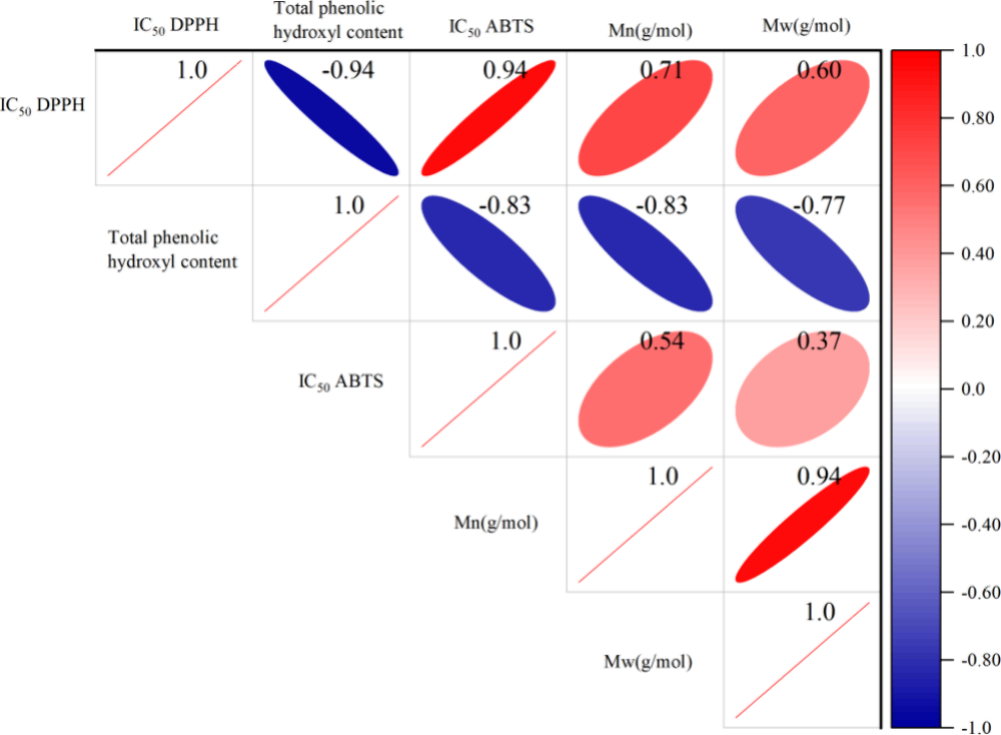
Correlation between total phenol content, antioxidant activity IC_50_ value, and molecular weight of the DES-extracted lignins.

In order to investigate the internal relationship between the chemical structure of DES-extracted lignins and their antioxidant capacity, the correlation between the total phenolic hydroxyl content and the IC_50 DPPH_ and IC_50 ABTS_ values was analyzed, and the results are shown in Figure 11. A strong negative correlation was observed between the total phenolic hydroxyl content of lignins and their scavenging ability for ABTS^+·^ and DPPH^·^, with *R*^2^ values of −0.83 and −0.94, respectively. This relationship is likely due to a proton-coupled electron transfer mechanism.^16^ These findings align with previous studies, which indicated the significant influence of total phenolic hydroxyl groups content on the antioxidant activity of lignin.^31^ You et al.^68^ treated alkali lignin with dilute acid and observed a significant increase in the total phenol content, with the phenolic hydroxyl content rising from 69.7 (mg GAE) /g lignin to 303.6 (mg GAE) /g lignin. Correspondingly, the IC_50 DPPH_ decreased from 173.4 μg/mL to 38.5 μg/mL. Similarly, Li et al.^69^ degraded alkaline lignin using acidic DESs and found a positive relationship between the phenolic hydroxyl group content and the antioxidant capacity of the regenerated lignin. Chai et al.^70^ modified alkaline poplar lignin with Lewis acid and found that the phenolic hydroxyl group content increased by 171.67%. Simultaneously, the antioxidant capacity of activated lignin against DPPH and ABTS free radicals was significantly increased by 2.58 and 4.64 times, respectively.

Molecular weight is also recognized as an important physicochemical property of lignin. Generally, lignins with lower molecular weights and higher phenolic hydroxyl group contents exhibit stronger antioxidant activities. As can be seen from Figure 11, there was a correlation between the molecular weight of DES-extracted lignins and their antioxidant activity, with *R*^2^ values of 0.71 and 0.60 for *M*_n_ and *M*_w_ with IC_50 DPPH_, and 0.54 and 0.37 for *M*_n_ and *M*_w_ with IC_50 ABTS_, respectively. Moreover, a strong negative correlation was observed between the total phenolic content of lignins and their *M*_n_ and *M*_w_ (*R*^2^= −0.83 and −0.77). This relationship may be due to the destruction of the β-O-4 bonds in the basic structural units of lignin during DES processing, reducing the degree of polymerization and molecular weight of lignin, increasing the formation of phenolic hydroxyl groups, and thereby endowing the lignin with superior antioxidant activity.^67,71^ Similarly, Liu et al.^72^ showed that the DPPH free radical scavenging activity of coffee bean lignin gradually increased as the molecular weight decreased. Xu et al.^73^ treated lignin with ChCl-Gly-AlCl_3_, and homogeneous lignins with molecular weights ranging from 1413 to 1812 g/mol were prepared, which showed excellent antioxidant capacity. Du et al.^74^ extracted lignin with lower molecular weight using a lithium bromide trihydrate system, achieving a maximum DPPH^·^ scavenging capacity with an IC_50_ value of 0.009 mg/mL (Table 5).

**Table 5:** Relationship between Phenolic Hydroxyl Content and IC_50_ Value of Six Kinds of DES-Extracted Lignins

lignin type	phenolic hydroxyl content (mg GEA/100 mg lignin)	*M*_n_ (g/mol)	*M*_w_ (g/mol)	IC_50_ DPPH (μg/mL)	IC_50_ ABTS (μg/mL)
ChCl-Lac lignin	119.081	846	2643	978.56	82.21
ChCl-HAc lignin	71.081	938	2573	1291.11	105.59
ChCl-FA lignin	144.172	601	1599	831.33	83.67
ChCl-MA lignin	121.142	736	1957	1061.64	98.21
ChCl-CA lignin	193.263	774	2258	582.95	61.75
ChCl-TsOH lignin	242.111	572	1590	417.69	58.62

Therefore, this research established a positive correlation between the antioxidant activity of the DES-extracted lignins and their phenolic hydroxyl content and a negative correlation with their molecular weight. This emphasizes the important influence of phenolic hydroxyl groups on the antioxidant activity of lignin, which is in agreement with previous studies.^75,76^

## Conclusions

In this study, the chemical structure and antioxidant activity of bamboo lignin extracted by six types of DESs prepared with choline chloride and various organic acids, including lactic acid, acetic acid, formic acid, malic acid, citric acid, and *p*-toluenesulfonic acid, were investigated. The main findings are as follows.

Organic acid-based DESs could realize the efficient extraction of bamboo lignin. Among them, ChCl-TsOH, ChCl-Lac, and ChCl-FA showed excellent delignification performance, with removal rates of 86.38%, 77.95%, and 75.28% respectively. The purity of lignin extracted by these DESs was high, ranging from 66.33% to 87.31%. FT-IR and UV–vis spectroscopy analyses confirmed that the extracted lignins were rich in hydroxyl, carboxyl, and carbonyl groups. Among the lignins extracted with the six DESs, ChCl-TsOH lignin, at a concentration of 0.2 mg/mL, showed the most effective shielding against both UV and visible light with a transmittance of only 0.074% at 400 nm. In addition, ChCl-CA lignin and ChCl-FA lignin also showed good UV shielding effects, with transmittances of 7.29% and 22.12% at 400 nm, respectively, at the same concentration. TG analysis revealed that the extracted-DESs lignins had a high thermal stability. Moreover, the DES-extracted lignins demonstrated good scavenging effects on DPPH and ABTS free radicals. The antioxidant activity of lignins was positively correlated to their phenolic hydroxyl content and negatively correlated to their molecular weight. Among the six DES-extracted lignins studied, ChCl-TsOH lignin showed the best antioxidant activity (IC_50 DPPH_: 417.69 μg/mL, IC_50 ABTS_: 58.62 μg/mL), the highest total phenolic hydroxyl content (242.11 mg GEA/100 mg lignin), and the lowest weight-average molecular weight (1590 g/mol) and number-average molecular weight (572 g/mol).

Therefore, ChCl-TsOH emerges as a potential DES for the pretreatment of lignocellulosic fiber materials. However, factors such as the ratio of HBAs to HBDs, concentration, treatment temperature, and time need to be considered when delignifying lignocellulosic materials using ChCl-TsOH, as these parameters can affect the yield of cellulose-rich materials and the purity of lignin. Unsuitable reaction conditions may lead to carbonization of the lignocellulosic biomass and hinder subsequent utilization. Future research should focus on optimizing these parameters not only to obtain lignin with high antioxidants but also to improve the retention of cellulose-rich materials, thus achieving an integrated and efficient utilization of lignocellulosic materials.
